# Histopathological Assessment of Dyspepsia in the Absence of Endoscopic Mucosal Lesions

**DOI:** 10.5005/jp-journals-10018-1178

**Published:** 2016-12-01

**Authors:** Hosam M Dawod, Mahmoud W Emara

**Affiliations:** 1 Department of Tropical Medicine, Faculty of Medicine, Zagazig University, Egypt; 2 Department of Pathology, Zagazig University, Egypt

**Keywords:** Biopsy, Chronic gastritis, Dyspepsia, Endoscopy.

## Abstract

**Introduction:**

Dyspepsia is a common symptom with an extensive differential diagnosis. Endoscopy alone may miss serious mucosal lesions in about 15 to 30% of cases. The aim was to determine histopathological features of gastric and duodenal mucosal biopsies in patients with dyspepsia and normal looking upper gastrointestinal (GI) endoscopy.

**Materials and methods:**

One hundred and five adult patients presenting with dyspepsia with no endoscopic mucosal lesions in the upper GI tract were included. Gastric biopsy specimens according to Sydney–Houston system for grading gastritis and biopsy from duodenum were taken. The histopathological features were graded according to the Sydney–Houston system classification for grading gastritis.

**Results:**

The histological lesions were found in 65.7% (69 out of 105 endoscopy free dyspeptic patients). Chronic inflammation was the commonest finding. Neutrophilic activity, glandular atrophy, and mild degree of intestinal metaplasia were present in 27, 45, and 6 patients (22.8, 42.8, and 5.7% respectively). *Helicobacter pylori* was present in 54 patients with histopathological lesions and in 6 patients without histopathological lesions, and the difference was significant (p = 0.045).

**Conclusion:**

The endoscopic diagnosis of dyspepsia correlated poorly with histopathological findings. The histopathological examination allowed detection and grading of gastric pathology in dyspepsia with normal endoscopy and the commonest finding was the moderate chronic gastritis.

**How to cite this article:**

Dawod HM, Emara MW. Histopathological Assessment of Dyspepsia in the Absence of Endoscopic Mucosal Lesions. Euroasian J Hepato-Gastroenterol 2016;6(2):97-102.

## INTRODUCTION

Dyspepsia is a term used for acute, chronic, or recurrent pain or discomfort centered in the upper abdomen. It may be associated with upper abdominal fullness, early satiety, and bloating, burning, belching, nausea, retching, and vomiting.^1^ Dyspepsia occurs in 25% of adult population and accounts for 5% of general clinics visits.^2^ Approximately 25% of patients with dyspepsia have an organic cause and up to 75% have functional dyspepsia.^3^

Patients with symptoms of dyspepsia who have not investigated are defined as having uninvestigated dyspepsia.^4^ In patients with dyspepsia who are investigated, major causes include medications, functional dyspepsia, chronic peptic ulcer disease, and malignancy.^2^ Other minor causes include pancreatic and hepatobiliary tract disease, motility disorders, infiltrative diseases of the stomach, celiac disease, metabolic disturbances, diabetic neuropathy, and hernia.^5^

The alarm signs, age, and the local prevalence of *Helicobacter pylori* infection determine the diagnostic approach of a case of dyspepsia.^6^ In patient with an age of ≤55 years, the American Gastroenterological Association (AGA) identifies unintended weight loss, progressive dysphagia, persistent vomiting, evidence of gastrointestinal (GI) bleeding, and family history of cancer as alarm signs. The AGA recommends endoscopy to be done first in patients with alarm signs and in patients >55 years.^1^ Patients ≤55 years of age without alarm features should be tested and treated for *H. pylori* if the local prevalence of *H. pylori* is high (>20%).^7^ Empiric acid suppressive treatments without *H. pylori* testing/treatment are recommended in areas of low prevalence for *H. pylori* (<20%).^2^

Once a patient has failed a 4 to 8 weeks trial of empiric treatment or failed to respond to eradication of *H. pylori*, upper endoscopy is indicated.^4^ Endoscopy alone is insufficient because it may miss serious mucosal lesions in about 15 to 30% of cases that can be picked up later on by histological examination.^8^ Biopsy is convenient procedure for accurate assessment and diagnosis of premalignant gastric lesions.^9^ Moreover, the biopsy is important for identifying and grading various mucosal pathologic lesions.^10,11^ The sites for biopsy taking from stomach according to the Sydney–Houston system are from the antrum, the angulus, and the corpus.^12,13^ The aim of this study was to determine histopathological features of gastric and duodenal mucosal biopsies in dyspepsia with normal looking upper GI endoscopy.

## MATERIALS AND METHODS

### Patient Selection

This study was conducted at the Department of Tropical Medicine, Faculty of Medicine, Zagazig University Hospitals in Egypt from July 2014 to August 2015, and 105 consecutive adult patients who were presented with dyspepsia and no mucosal lesions by the upper endoscopy were selected.

Exclusion criteria: Recent history of nonsteroidal anti-inflammatory drugs (NSAIDs), proton pump inhibitors, antibiotic intake within 1 month, peptic ulcer disease, gastroesophageal (GE) reflux disease, choledocholithiasis, pancreatitis, uncontrolled diabetes mellitus, hypercalcemia, coagulopathy, alcohol intake, celiac disease, chronic disease, end organ failure, immunocompromised, abdominal malignancy, or gastrostomy. This study was carried out in accordance with the World Medical Association Code of Ethics (Declaration of Helsinki) and a written informed consent for endoscopy, biopsy, and for taking blood sample was obtained from all patients. The protocol was approved by a review board of Tropical Medicine Department of Zagazig University.

### Study Design

A cross-sectional study was conducted on 105 patients presenting with dyspepsia, with no mucosal lesions were found in the upper GI tract by endoscopy. Patients were further evaluated by obtaining gastric biopsy specimens from the following sites according to Sydney–Houston system for grading gastritis: (1) Greater and lesser curvature of the distal antrum, (2) greater and lesser curvature of the proximal corpus, and (3) lesser curvature at the incisor angularis.^14^ One biopsy from the duodenum was also taken by standard biopsy forceps. The specimens were properly labeled, fixed in 10% buffered formalin, processed using paraffin embedding technique, sectioned at 4 μm perpendicular to the mucosal surface and stained with hematoxylin and eosin (H&E) and with Giemsa. The same pathologist examined all the materials. The histopathological features were graded.

All patients in this study were subjected to history taking, clinical examination, complete blood picture, fasting blood sugar, random blood sugar, blood urea and serum creatinine, liver function tests, *H. pylori* antigen in stool testing, and abdominal ultrasonography. Upper GI endoscopy was done after overnight fast for 6 hours using Pentax EPM-3500; Tokyo, Japan. The endoscopic examination was done by the same endoscopist.

### Statistical Analysis

Data were coded and analyzed using Statistical Package for the Social Sciences (SPSS) version 16. Data were expressed as mean ± standard deviation (SD) for quantitative variables, number, and percentage for qualitative ones. Chi-squared and McNemar tests were used when appropriate, with p < 0.05 considered as statistically significant. The sample size was calculated using Epi-Info version 6 at power of study, 80 and 95% confidence interval, and the level of significance < 0.05.

## RESULTS

A total of 105 patients (51 males and 54 females; mean age 45.54 ± 9.5, range: 22–60 years) who presented with dyspepsia and no mucosal lesions found in the upper GI tract by endoscopy were enrolled in the study (Table 1).

**Table 1:** **Table 1:** Demographic characteristics of the patients

*Data*		*n = 105 patients*	
Gender (%)			
Male		51 (48. 6%)	
Female		54 (51.4%)	
Age (years)			
Mean ± SD		45.54 ± 9.50	
Range		22–60	
Frequency of histopathological lesions in dyspeptic patients		69 (65.7%)	

The laboratory parameters of the patients were within normal values (Table 2). Epigastric pain and epigastric burning were the most common presentation of the studied patients (45 and 27 patients) (42 and 25.7%) respectively, while dysphagia and nausea in 6 and 3 patients (5.7 and 2.9% respectively) were the least common presentations (Table 3).

**Table 2:** **Table 2:** Laboratory data of studied patients

*Variable*		*Mean ± SD*		*Range*	
WBC (×10^3^/mL)		7.29 ± 4.62		4.5–11	
Hemoglobin (mg/dL)		13.05 ± 1.60		8–15	
Platelet (×10^3^/mL)		274.11 ± 82.27		156–411	
ALT (IU/mL)		17.6 ± 4.50		12–24	
AST (IU/mL)		19.00 ± 4.62		12–30	
Bilirubin (mg/dL)		0.74 ± 0.17		0.5–1.2	
Albumin (mg/dL)		4.19 ± 0.27		3.8–5	
INR		1.15 ± 0.061		1.1–1.3	
Creatinine (mg/dL)		0.7 ± 0.12		0.8–1.1	

ALT: Alanine aminotransferase; AST: Aspartate aminotransferase; INR: International normalized ratio

**Table 3:** **Table 3:** Clinical presentation of studied patients

*Symptom*		*Number*		*Percentage*	
Epigastric pain		45		42.8	
Epigastric burning		27		25.7	
Unexplained weight loss		15		14.3	
Persistent vomiting		9		8.6	
Dysphagia		6		5.7	
Nausea		3		2.8	
Total		105		100	

**Graph 1:**
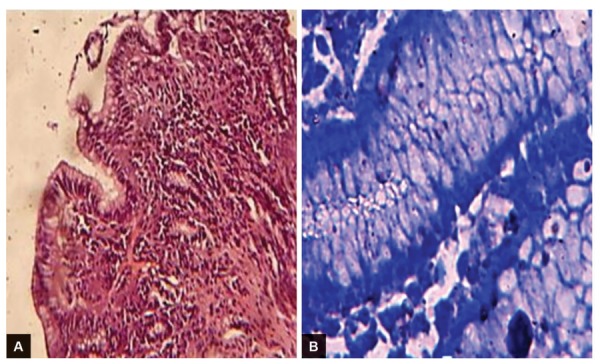
Pathological variability in patients with functional dyspepsia

**Figs 1A and B:**
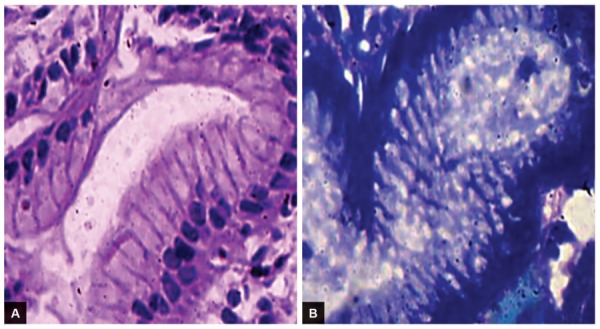
Chronic gastritis and positive *H. pylori* infection: (A) H&E stain; and (B) geimsa stain

**Figs 2A and B:**
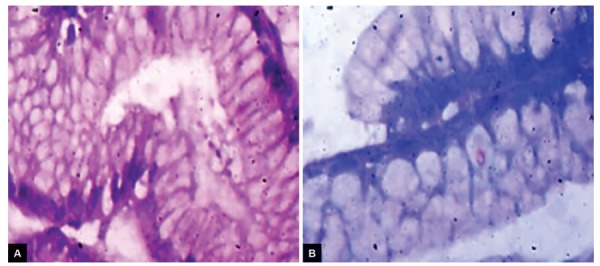
Chronic gastritis and positive *H. pylori* infection: The lamina propria is moderately infiltrated by chronic inflammatory cells, mainly plasma cells and lymphocytes with few neutrophils: (A) H&E stain; and (B) geimsa stain

**Figs 3A and B:**
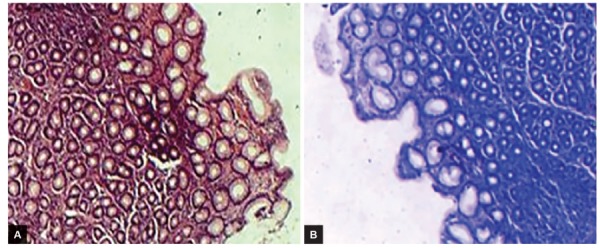
Chronic gastritis, positive *H. pylori* infection, and intestinal metaplasia: (A) H&E stain; and (B) geimsa stain

The frequency of histological lesions among dyspeptic patients was 65.7% (69 out of 105 patients), while no lesions in the other 36 (34.3%) patients were observed by endoscopy and biopsy (Table 2). The commonest lesion was the chronic inflammation of gastric mucosa (65.7%) (Table 4, Graph 1). The grading of the chronic inflammation was as follows: 24 patients with mild chronic inflammation, 39 patients with moderate chronic inflammation (Figs 1A and B), and 6 patients with marked chronic inflammation. Neutrophilic activity was present in 27 patients with only 3 patients with marked neutrophilic activity (Figs 2A and B). *Helicobacter pylori* density was present in 54 patients and 30 of them showed mild degree of *H. pylori* density. Glandular atrophy was present in 45 patients with only 6 patients with marked degree of glandular atrophy. Mild degree of intestinal metaplasia was only present in 6 female patients (Figs 3A and B).

**Table 4:** **Table 4:** Pathological variability in patients with dyspepsia

		*Grade*									
*Variability*		*None*		*Mild*		*Moderate*		*Marked*		*Total n (%)*	
Chronic gastritis		36		24		39		6		69 (65.7)	
Neutrophilic activity		78		9		15		3		27 (22.8)	
H. pylori density		51		30		12		12		54 (51.4)	
Glandular atrophy		60		18		21		6		45 (42.8)	
Intestinal metaplasia		99		6		–		–		6 (5.7)	

**Figs 4A and B:**
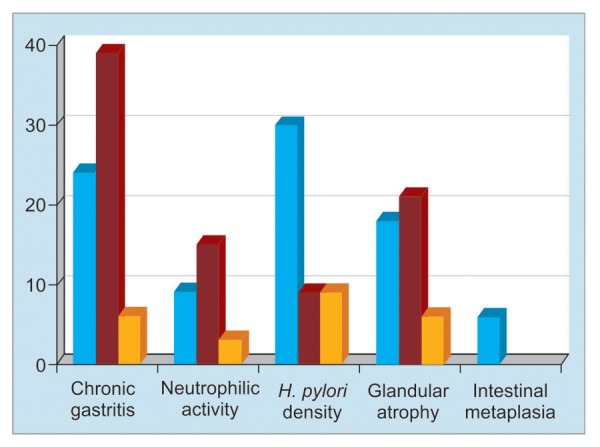
Chronic gastritis and negative *H. pylori* infection: (A) H&E stain; and (B) geimsa stain

Table 5 shows that the *H. pylori* infection was present in 6 patients without histopathological lesion (16.67%) (Figs 4A and B), while it was present in 54 patients with histopathological lesion (78.2%) and the difference was statistically significant (p = 0.04). *Helicobacter pylori* infection was present in 24 patients (53.33%) out of 45 patients with glandular atrophy. All the patients tolerated endoscopy and biopsy with no complications.

**Table 5:** **Table 5:** Distribution of *H. pylori* infection in dyspeptic patients with and without histopathological lesion

*H. pylori*		*Without HPL*		*With HPL*		*Total*		*p-value*	
+ve		6 (16.67%)		54 (78.2%)		60			
–ve		30 (83.33%)		15 (20.8%)		45			
Total		36		69		105		0.04*	

*p & 0.05: Significant; HPL: Histopathological lesion

## DISCUSSION

Dyspepsia is a common symptom and represents a great health care problem.^15^ Around 60% of patients with dyspepsia still have no explanation for their symptoms.^16^ The aim of this study was to determine histopathological features of gastric and duodenal mucosal biopsies in dyspeptic patients with free endoscopy. In this study, the histopathological examination of the gastric biopsies showed normal findings in around 34.3% and a high degree of inflammation which occurred in 65.7%. This result was in agreement with observations by Nwokediuko and Okafor,^17^ who found that only 29.3% of patients with functional dyspepsia had normal histology of gastric biopsies by histological examination. This result is also in accordance with which was reported by Arruda et al,^18^ who studied 40 dyspeptic patients (28 women and 12 men) with endoscopically normal stomach and found 72.5% of patients with gastritis by histological examination. This emphasizes the role of biopsy in examining a case of dyspepsia as the normal endoscopy does not rule out underlying pathology. Garg et al^19^ and Khan et al^20^ reported in their studies a lower rate of inflammation in the gastric biopsies (20 and 32% respectively) of dyspeptic cases with a normal endoscopy. This difference may be attributed to high prevalence of *H. pylori* infection among Egyptian patients.

Chronic inflammation was present in 65.7% of patients with dyspepsia and was higher than that of *H. pylori* infections (51.4%). This could be explained by the presence of other causes of inflammation than *H. pylori* or previous ingestion of antibiotics which are known to suppress the *H. pylori* infection with a slow disappearance of chronic inflammatory cells.^21^

In the present study, the prevalence of *H. pylori* was 51.4%. Nwokediuko and Okafor^17^ and Calabrese et al^22^ reported lower rates of the prevalence of *H. pylori* (37.3 and 30.9% respectively) in patients with normal looking mucosa, while Shrivastava et al^23^ observed a higher incidence rate of *H. pylori* infection among functional dyspeptic patients (65%). This could be explained by variations in the prevalence of *H. pylori* according to geographic area, age, and socioeconomic status.^24^ Moreover, there is a variation in diagnostic methods which may further contribute to different rates of detection.

A highlight on the relation between *H. pylori* infection and chronic gastritis was noted in our study, as *H. pylori* was present only in 6 patients without histopathological lesions, while it was present in 54 patients with histopathological lesions and the difference was statistically significant (p = 0.04), which is matching with the fact that the main cause of chronic gastritis is *H. pylori* infection.^25^

Intestinal metaplasia and glandular atrophy were present in 5.7 and 42.8% respectively, of patients with dyspepsia. However, these results were less than those reported by Leodolter et al^26^ and Anatoliy et al^27^ (24.1 and 64.0% respectively), and this difference may be due to including patients with functional dyspepsia and positive *H. pylori* infection in their studies.

Biopsy was not taken from the lower esophagus as patients with history or symptoms of reflux disease were not included and this is in accordance with the recent recommendation of AGA^28^ that routine biopsy of normal esophagus or GE junction in patients with dyspepsia alone would have very low probability of diagnosing esophageal abnormalities or have little impact on clinical management. Piatek et al^11^ advised biopsy taking from stomach and duodenum only in either organic or functional dyspepsia. They reported in their study nonspecific inflammation of all normal looking duodenums independent of *H. pylori* state, unlike in our study where the duodenal biopsy showed nonspecific inflammation in 3 patients only. Our results clarify the need to obtain gastric biopsies during upper GI endoscopy even if the endoscopic appearance is normal or without any gross pathology which is matching with recent recommendations from AGA.^28^

## CONCLUSION

The conventional endoscopic diagnosis of dyspepsia correlated poorly with histopathological findings. The histopathological examination allowed detection and grading of gastric pathology in dyspepsia with normal endoscopy and it showed that the moderate chronic gastritis was the most common finding in this study.
